# Assembly of the *Boechera retrofracta* Genome and Evolutionary Analysis of Apomixis-Associated Genes

**DOI:** 10.3390/genes9040185

**Published:** 2018-03-28

**Authors:** Sergei Kliver, Mike Rayko, Alexey Komissarov, Evgeny Bakin, Daria Zhernakova, Kasavajhala Prasad, Catherine Rushworth, R. Baskar, Dmitry Smetanin, Jeremy Schmutz, Daniel S. Rokhsar, Thomas Mitchell-Olds, Ueli Grossniklaus, Vladimir Brukhin

**Affiliations:** 1 Dobzhansky Center for Genome Bioinformatics, St. Petersburg State Universit, Sredniy Prospekt, 41, Vasilievsky Island, 199004 St. Petersburg, Russia; mahajrod@gmail.com (S.K.); mikerayko@gmail.com (M.R.); ad3002@gmail.com (A.K.); eugene.bakin@gmail.com (E.B.); dashzhernakova@gmail.com (D.Z.); 2 All-Russia Research Institute for Agricultural Microbiology, Podbelskogo sh. 3, Pushkin, 196608 St. Petersburg, Russia; 3 Department of Biology, Colorado State University, Fort Collins, CO 80523; USA; kasavajhalaprasad@gmail.com; 4 University and Jepson Herbaria, University of California, Berkeley, NC 94720; USA; crushworth@berkeley.edu; 5 Department of Biotechnology, Indian Institute of Technology. Sardar Patel road, 600036 Chennai, India; rbaskar@iitm.ac.in; 6 Department of Plant and Microbial Biology Zurich-Basel Plant Science Center, University of Zurich, Zollikerstrasse 107, 8008 Zurich; Switzerland; dmitry.smetanin@botinst.uzh.ch (D.S.); grossnik@botinst.uzh.ch (U.G.); 7 Department of Energy Joint Genome Institute, Walnut Creek, CA 94598; USA; jschmutz@hudsonalpha.org (J.S.); dsrokhsar@gmail.com (D.S.R.); 8 HudsonAlpha Institute of Biotechnology, Huntsville, AL 35806; USA; 9 Department of Biology, Duke University, Durham, NC 27708-0338; USA; tmo1@duke.edu; 10 Department of Plant Embryology and Reproductive Biology, Komarov Botanical Institute RAS, 197376 St. Petersburg, Russia

**Keywords:** *Boechera*, Brassicaceae, genome, assembly, annotation, apomixis

## Abstract

Closely related to the model plant *Arabidopsis thaliana*, the genus *Boechera* is known to contain both sexual and apomictic species or accessions. *Boechera retrofracta* is a diploid sexually reproducing species and is thought to be an ancestral parent species of apomictic species. Here we report the de novo assembly of the *B. retrofracta* genome using short Illumina and Roche reads from 1 paired-end and 3 mate pair libraries. The distribution of 23-mers from the paired end library has indicated a low level of heterozygosity and the presence of detectable duplications and triplications. The genome size was estimated to be equal 227 Mb. N50 of the assembled scaffolds was 2.3 Mb. Using a hybrid approach that combines homology-based and de novo methods 27,048 protein-coding genes were predicted. Also repeats, transfer RNA (tRNA) and ribosomal RNA (rRNA) genes were annotated. Finally, genes of *B. retrofracta* and 6 other Brassicaceae species were used for phylogenetic tree reconstruction. In addition, we explored the histidine exonuclease *APOLLO* locus, related to apomixis in *Boechera*, and proposed model of its evolution through the series of duplications. An assembled genome of *B. retrofracta* will help in the challenging assembly of the highly heterozygous genomes of hybrid apomictic species*.*

## 1. Introduction

Among over the 370 genera belonging to the family Brassicaceae (Cruciferae), only the genus *Boechera* shows asexual reproduction by seeds [1,2,3,4]. Apomixis is defined as asexual reproduction through seeds that results in progeny identical to the maternal plant. The harnessing of apomixis is widely considered as a key enabling technology for crop improvement because it allows the fixation of any heterozygous genotype, leading to simpler and faster breeding schemes [5,6,7]. The *Boechera* genus includes 110 sexual and apomictic species, widely distributed in North America. Plants from the *Boechera* genus are represented by biannual and perennial herbs with a chromosome base number of *n* = 7 [8,9].

Apomixis in the *Boechera* genus is of special interest because it can occur at the diploid level, which is very rare [1,2,3,4,5,6,7,8]. Furthermore, the phylogenetic proximity of *Boechera* to the model plant *Arabidopsis thaliana* is attractive for potential functional studies. Although the genus *Boechera* includes both sexual and apomictic species and accessions that are of variable ploidy and geographical origin, search for homologous sequences are feasible across the genus [10]. The sexual accessions of *Boechera* are self-compatible and largely self-pollinating [11], unlike the sexual ancestors of most other apomicts, which are typically self-incompatible and cross-pollinating [12]. This inbreeding causes low heterozygosity in sexual *Boechera* species. Apomictic *Boechera* accessions have likely arisen through independent hybridization events [13]. Their hybridogenic origin is supported by the aberrant structure of their chromosomes, as they are often observed as a consequence of hybridization, leading to alloploidy, aneuploidy, the replacement of homeologous chromosomes, and aberrant chromosomes [13,14].

**Figure 1:**
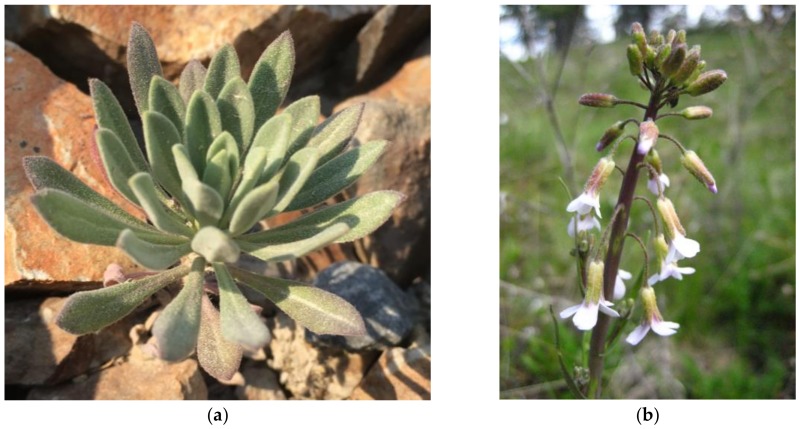
Plant (**a**) and flower (**b**) of *Boechera retrofracta*.

Certain apomictic *Boechera* accessions are hypothesized to have arisen through hybridization between sexual *Boechera stricta* and *Boechera retrofracta* (Figure 1). *Boechera retrofracta* was previously included within *Boechera holboellii* (sensu lato) [15]. Up to now only the genome sequence of *B. stricta* was available [16], while the genome of *B. retrofracta* has not been assembled yet.

In this paper, we present the assembly and annotation of the *B. retrofracta* genome. The availability of the *B. retrofracta* genome sequence together with the previously assembled *B. stricta* genome will greatly help in the assembly and annotation of related apomictic hybrid species and provide the basis to investigate the peculiarities of hybridization events, chromosomal organization, the stability of apomictic genomes, and the genetic factors underlying apomixis. The performed assembly and annotation allowed us to analyze of the *APOLLO* (APOmixis-Linked LOcus) genes, that are associated with apomixis in *Boechera*.

## 2. Materials and Methods 

### 2.1. Sample Information 

The reference *B. retrofracta* genotype was collected in Panther Creek, Lemhi County, Idaho, at 45°18′11.9″ N 114°22′35.9″ W, 1610 m elevation (Figure 1). Plant growth, DNA extraction, and library construction were the same as with *B. stricta* [16]. Briefly, seedlings were germinated in aseptic culture in half-strength Murashige and Skoog (MS) liquid medium. Cell nuclei were used for isolation of clean high-molecular-size nuclear DNA in Tris-EDTA (TE) buffer. 

### 2.2. Sequencing Strategy

The *B. retrofracta* genome was sequenced within the JGI Community Sequencing Project to produce sequence data for the *Boechera* genus [17]. Six libraries were constructed and sequenced using three platforms including Illumina, Roche, and Sanger: one paired-end (PE) library, four mate pairs (MP) libraries and one Sanger bacterial artificial chromosome (BAC) end library. Read length and actual insert sizes for each library are given in Appendix A (Table A1). This sequencing scheme was specially developed for the initial contig assembly by the DISCOVAR assembler [18], followed by scaffolding. Construction of genomic libraries and sequencing were performed following Lee et al [16].

### 2.3. Raw Data Filtration and Pre-Processing

Filtration of the PE library LIB400 was performed in two stages. First, reads containing long adapter fragments were removed using Cookiecutter [19]. Then Trimmomatic [20] was used to filter out reads with short adapter fragments. However, according to the DISCOVAR requirements no trimming or quality filtration was performed at those two stages. Only whole reads contaminated by adapters were discarded.

To process Illumina MP libraries LIB5000 and LIB7000 the NextClip [21] tool was modified to handle Cre-Lox libraries. It is important to note that original NextClip uses a very simple algorithm to align linker sequences to reads. It takes into account only the number of matching bases. As the CreLox linker is significantly longer than the Nextera linker, the number of false hits may significantly increase. To mitigate this effect, a requirement for the presence of a continuous 9-bp core alignment was added. The modified tool was named CreClip and can be found in [22].

Reads from Roche MP Libraries LIB4000R and LIB24000R were split into “forward” and “reverse” segments separated by linker. Then, low quality ends were trimmed from “reverse” segments by Trimmomatic. Finally, reverse segments were reverse complemented to mimic to Illumina MP libraries.

### 2.4. Genome Size Estimation

Estimation of the genome size based on the 23-mer distribution (as well as other *k*-mer based statistics) was performed using the KrATER software [23] on the LIB400 library and further compared with the previous estimations of *Boechera* genus [24].

### 2.5. Genome Assembly and Quality Evaluation

At the assembly stage initial contigs were constructed from the filtered LIB400 reads by DISCOVAR. Then, the obtained contigs were extended using a BAC end sequencing (BES) library and the SSPACE scaffolder [25].

**Figure A1:**
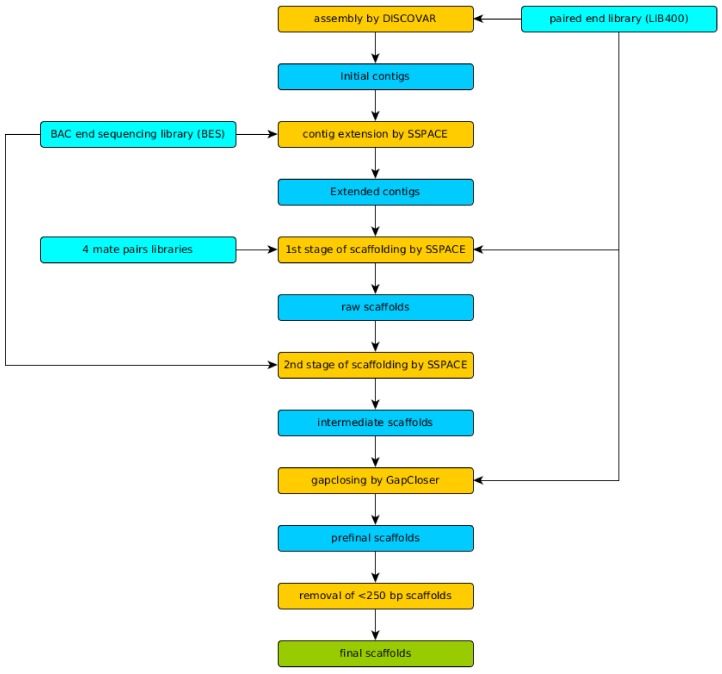
Pipeline used to assembly genome of *Boechera retrofracta*.

Before scaffolding the assessment of the actual (mean) insert size was performed. Filtered reads from all libraries were aligned to initial contigs by Burrows–Wheeler Aligner (BWA) [26]. For each library, only alignments to contigs with 3× length of the target insert size were used in the estimation (Table 1) to minimize alignment artifacts. Next, the extended contigs were scaffolded by SSPACE in two stages: at the first stage, all four MP libraries (LIB4000R, LIB5000, LIB7000, LIB24000R) were used to produce raw scaffolds, at the second stage, raw scaffolds were linked to the intermediate scaffolds using the BES library only. Scaffolding was carried out in several stages because different options were required to utilize the BES data. Gap closing in the intermediate scaffolds was performed using GapCloser (a module for SOAPdenovo2) [27] on the LIB400 library only. Finally, all scaffolds with a length of less than 250 bp (i.e., less than read length of LIB400, the library used for initial contig construction) were filtered out, as the corresponding short fragments most likely are the assembly artifacts. Integrity of the assembly was verified by Core Eukaryotic Genes Mapping Approach (CEGMA) [28] and Benchmarking Universal Single-Copy Orthologs (BUSCO) [29]. A schematic diagram of the assembly pipeline is shown in Figure A1 in Appendix A.

### 2.6. Repeats Analysis

A de novo repeat identification in the *B. retrofracta* genome was performed using RepeatModeler [30] with default parameters. The obtained repeat library was combined with *Arabidopsis thaliana* repeats from RepBase [31], and the merged library was used to annotate repeats by RepeatMasker [32]. Then repeats in the *B. retrofracta* genome were softmasked by Bedtools [33] for the prediction of protein coding genes. Also, masking of tandem and interspersed repeats by tandem repeats finder (TRF) [34] and WindowMasker [35], respectively, were performed.

### 2.7. Variants Calling and Genotyping

For variant calling and genotyping filtered reads were aligned to the assembled genome using BWA mem with default options. Next, the Genome Analysis Toolkit (GATK) pipeline [36] for variant calling was applied in the following way: duplicates were marked using Picard MarkDuplicates (Broad Institute, Cambridge, MA, USA), realigned reads at indels, and, finally, HaplotypeCaller (Broad Institute) was used to call variants. Only single nucleotide polymorphisms (SNPs) and indels were kept passing the following filtering criteria: QualByDepth (QD) > 2.0, FisherStrand (FS) < 20.0, RMSMappingQuality (MQ) > 40.0, MappingQualityRankSumTest (MQRankSum) > −12.5, ReadPosRankSumTest (ReadPosRankSum) > −8.0 for SNPs, and QualByDepth (QD) > 2.0, FisherStrand (FS) < 20.0, ReadPosRankSumTest (ReadPosRankSum) > −20.0 for indels, respectively. Finally, the variants falling into the repeats masked by RepeatMasker were excluded.

### 2.8. Prediction of Protein-Coding Genes and Non-Coding RNA

The prediction of protein-coding genes was performed using a combined approach that synthesizes both homology-based and de novo predictions, where de novo predictions are used only to fill gaps and to extend the homology-based predictions. Pure de novo predictions were filtered out.

As homology-based evidence for gene presence, we have used proteins and transcripts of five closely-related species. Proteins of the four reference species—*Arabidopsis thaliana* (assembly TAIR10), *Brassica rapa* (Brapa_1.0), *Capsella rubella* (Caprub1_0), and *Eutrema salsugineum* (Eutsalg1_0)—were aligned to the *B. retrofracta* assembly by Exonerate [37], using the Protein2Genome model with a maximum of three hits per protein. The obtained alignments were classified into the top (primary) and secondary hits; the coding sequence (CDS) fragments were cut from each side by 3 bp for the top hits and by 9 bp for the secondary hits. Transcripts of *B. stricta* (assembly v1.2, [16]) with marked CDS regions were also aligned to the *B. retrofracta* genome by Exonerate using the cDNA2Genome model leaving the other options unchanged. Alignments of CDS segments were not cut for top hits, but cut by 3 bp for secondary hits. 

These truncated fragments were clustered and supplied as hints to the AUGUSTUS software package [38], and the CDS segments of genes were predicted in the soft-masked *B. retrofracta* assembly using *A. thaliana* gene models. Proteins were translated from the predicted genes and aligned by HMMER 3.1 [39] and BLAST [40] to the Pfam [41] and Swiss-Prot [42] databases, respectively. Only genes supported by the both hints and hits to one of the protein databases were retained; the rest were discarded. Transfer RNA (tRNA) and ribosomal RNA (rRNA) genes were predicted by tRNAscan-SE v1.3.1 [43] and Barrnap v0.6 [44], respectively.

### 2.9. Phylogenetic Analysis

The longest proteins corresponding to each predicted gene of *B. retrofracta* and six other Brassicaceae species—*B. stricta* (assembly v1.2), *A. thaliana* (TAIR10), *Arabidopsis lyrata* (v.1.0)*, Capsella rubella (Caprub1_0), Cardamine hirsuta* (v1.0), and *Eutrema salsugineum* (Eutsalg1_0)—were aligned to profile Hidden Markov Models (HMM) of the braNOG subset from the eggNOG database [45] using HMMER. The top hits from the alignments were extracted and used for assignment of the corresponding proteins to orthologous groups, followed by extraction of single-copy orthologs.

To verify topology concordance and get a basis for future studies of positive selection, a species tree reconstruction was performed. Single-copy orthologous proteins of the seven species included in the analysis were aligned by multiple alignment using fast Fourier transform (MAFFT) [46]. Based on the obtained protein alignments, a maximum likelihood tree was reconstructed by RAxML v8.2 [47] with the PROTGAMMAAUTO option, and the JTT fitting model was tested with 1000 bootstrap replications. The tree was rooted with *E. salsugineum* as an outgroup. The resulting tree was drawn with FigTree software [48].

### 2.10. APOLLO Evolution Analysis

The evolutionary history of *APOLLO* gene was inferred by using the Maximum Likelihood method. Initial alignment of corresponding CDS was performed using prank v.140110 [49] in codon-aware mode. The alignment result was further used for building phylogenetic tree basing on the Tamura-Nei model [50,51]. The tree with the highest log likelihood (−12,153.79) was selected (see Section 3.7, Figure 4). Initial tree(s) for the heuristic search were obtained automatically by applying Neighbor-Join and BioNJ algorithms to a matrix of pairwise distances estimated using the maximum composite likelihood (MCL) approach, and then selecting the topology with superior log likelihood value. All positions containing gaps and missing data were eliminated. There were a total of 1158 positions in the final dataset. Evolutionary analyses were conducted in MEGA7 [52].

### 2.11. Whole-Genome Comparison

As a preliminary step for the future whole-genome comparison of different *Boechera* species a whole genome alignment was performed via Cactus multiple genome aligner [53] and further visualized with web-tool ClicO FS [54] based on Circos [55].

For further information about the initial data and results, see Appendix B.

## 3. Results

### 3.1. k-mer Based Statistics 

**Figure 2:**
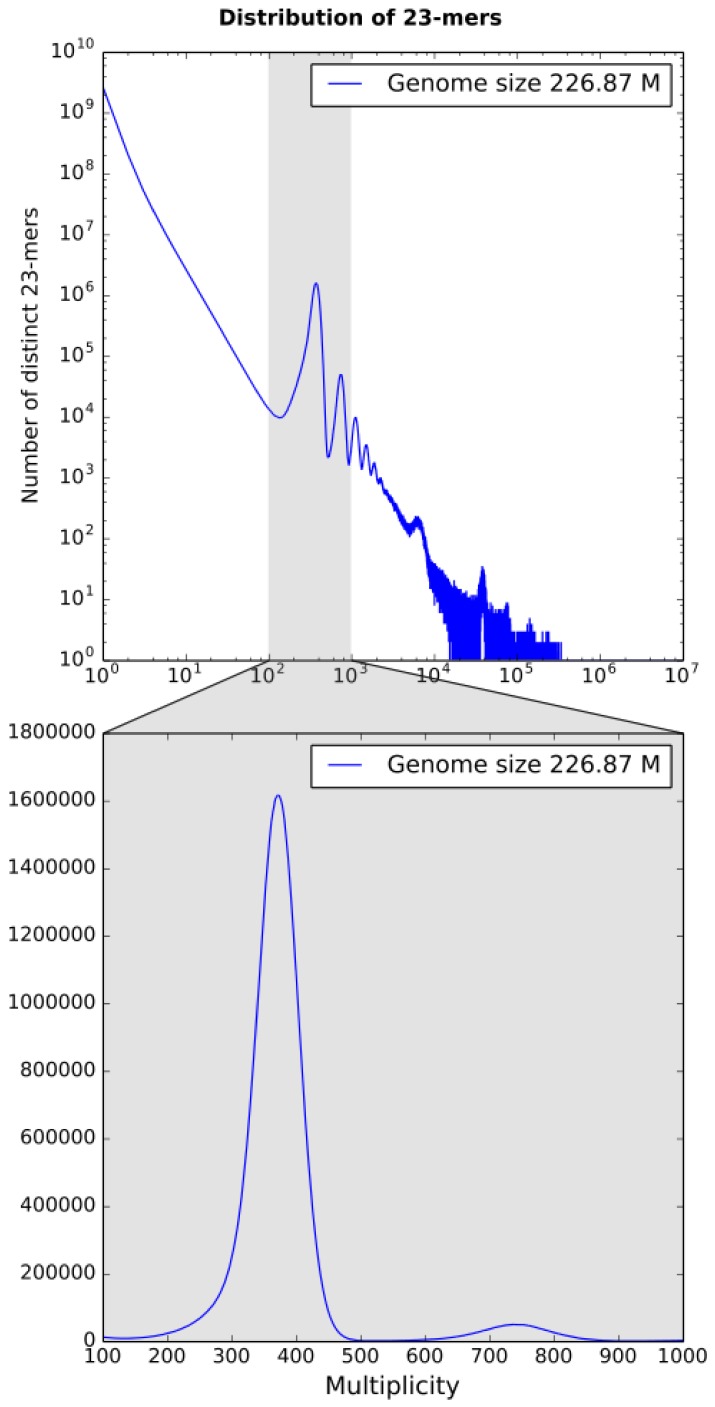
Distribution of 23-mers for PE LIB400 library. Only one major peak at 371× coverage is present, however there are detectable duplications and triplications at 737× and 1120× coverage (upper plot, Y axis is on a logarithmic scale).

*k*-mer spectrum built by KrATER [23] is shown in Figure 2. The 23-mer distribution has a peak of erroneous 23-mers at 1× coverage corresponding to sequencing errors and one major peak at 371× coverage corresponding to diploid 23-mers (shared between homologous chromosomes), but no significant peak related to heterozygous genome positions was detected (Figure 2). However, we detected several small additional peaks at double (737×) and triple (1120×) depth, which are probably related to duplications and triplications, respectively.

The genome size of *B. retrofracta* was estimated to be close to 227 Mbp, which is close to the previous estimations of a minimal genome size of 200 Mbp in the *Boechera* genus [24].

### 3.2. Genome Assembly and Evaluation 

We have achieved N50 of 2,297,899 bp, L50 of 25, and a total assembly length of 222.25 Mbp for the final scaffolds, which is very close to our 23-mer based estimation. Detailed statistics including N50 and total assembly values for every stage of the assembly pipeline are listed in Table 1 and Table 2. It is important to note that the final assembly (Table 1, column final scaffolds) has smaller size than previous intermediate assemblies due to the last filtration step. All scaffolds shorter than 250 bp (a read length of LIB400) were treated as artifacts of assembly and were removed. However, size of final assembly (222.25 Mbp) is closer to estimated genome size (226.87 Mbp) than the size of intermediate assemblies.

Evaluation of the assembly completeness was performed using CEGMA [28] and BUSCO [29]. In the assembled genome 242 (97.58%) complete core eukaryotic genes (CEGs) were identified. Out of 1440 BUSCO genes from the Embryophyta, set only 12 (0.8%) genes were not found, 6 were fragmented, 36 (2.5%) were duplicated and 1422 (98.8%) were complete. This high fraction of complete BUSCO genes suggests high completeness of the assembly and its integrity at least in gene-coding regions.

### 3.3. Repeats Annotation

In total approximately 85 Mbp (38.13%) of the assembly were masked. The detailed description of the annotated repeat types is listed in Table 3. It is important to note that a large number (10.96% of the assembly size) of interspersed repeats was not classified. The results are shown in Table 4.

### 3.4. Variant Calling and Genotyping

In the genome 3341 SNPs and 1317 indels were detected. Among these, 103 (3.08%) SNPs and 97 (7.37%) indels were homozygous and, therefore, most likely artifacts of alignment or assembly or SNP calling. Mean heterozygous SNP and indel densities in non-masked regions (138 Mbp in total) are 0.0235 SNP and 0.0089 indel per Kbp, respectively, suggesting a very low heterozygosity of the *B. retrofracta* genome.

### 3.5. Prediction of Protein-Coding Genes and Non-Coding RNAs 

In total 27,048 genes with 28,269 transcripts were predicted. tRNA and rRNA genes predicted by tRNAscan-SE and Barrnap are given in Table 5 and Table 6 respectively. 

### 3.6. Species Tree Reconstruction

In course of the assignment of proteins to orthologous groups 8959 single-copy orthologs were identified among the seven species (*B. retrofracta*, *B. stricta*, *A. thaliana*, *A. lyrata*, *C. rubella, C. hirsuta*, and *E. salsugineum*). 

**Figure 3:**
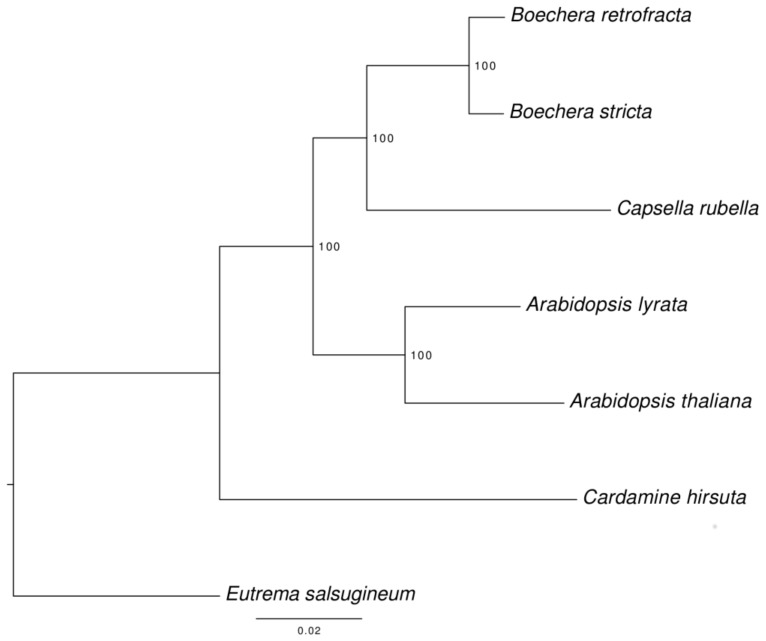
Phylogenetic tree of seven Brassicaceae species used for analysis. Maximum likelihood tree was reconstructed by RAxML using 8959 single copy orthologs and was tested with 1000 bootstrap replicates. Numbers near nodes represent corresponding bootstrap support.

The corresponding phylogenetic tree was rooted with *E. salsugineum* as an outgroup (Figure 3). All nodes have a high support and no topology discordance was found with the tree reconstructed previously by Huang et al [52].

### 3.7. Analysis of Evolution of the APOLLO Locus

Results from Corral et al. [56] suggest that *APOLLO* (aspartate glutamate aspartate aspartate histidine exonuclease) is one of the important apomixis-related genes in *Boechera*. It was shown that the *APOLLO* locus has several alleles with apomixis-associated polymorphisms. All studied apomictic plants carry at least one of the “apoalleles”, while both copies in sexual genotypes were “sexalleles”.

**Figure 4:**
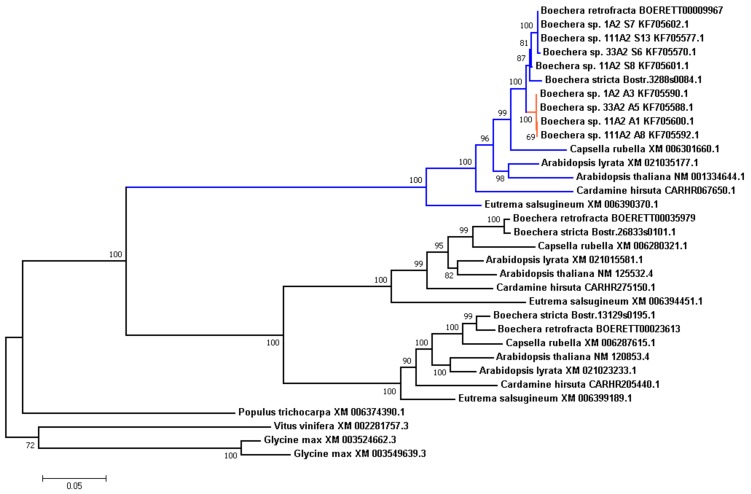
Phylogenetic tree of the isoforms of *APOLLO* locus (exonuclease NEN) in seven species of interest and alleles of *APOLLO* locus of apomictic *Boechera* species from Corral et al (2013) [56]. Sequences of *Populus trichocarpa*, *Vitus vinifera* and *Glycine max* were used as outgroup. The clade related to the *APOLLO* locus is shown in green, with apo-alleles shown in red. Numbers near nodes represent corresponding bootstrap support.

In this study we decided to take a closer look to this locus in our assembly and other Brassicaceae species in this study. Along with an exact copy of the *APOLLO* locus, we also found two other, more distant copies, which may indicate past duplication events. We searched for these orthologs in other species, and reconstructed phylogenetic tree (Figure 4). All Brassicaceae genomes in the study also carried these three copies, related to the clusters of orthologous genes ENOG410BURN (*APOLLO* locus), ENOG410BUTR, and ENOG410C333 in the EggNOG database.

We observed that branches in the tree were grouped by genes rather than by species, suggesting that the triplication event took place before the separation of the Brassicaceae species in this study. It is worth noting that in *Populus trichocarpa* genome there is only one copy of these locus, which gives an upper-bound time estimate of the series of duplication events. 

We also examined *APOLLO* alleles (both apo- and sex-alleles) described in *Boechera ssp*. by Corral et al. [56]. We can see that these alleles arise after the separation of the *Boechera* genus, and compose two separate clades. Given the fact that *B.retrofracta* and *B.stricta* are the sexual species, it was not surprising that in both cases all corresponding polymorphic sites were in the “sexallele”-state, and clustered with sex-alleles.

We calculated the Ka/Ks ratio for the internal branches in this tree and found that branch leading to apo-alleles is under positive selection (Ka/Ks 1.4646, the branch is shown in red in Figure 4), which is typical for paralogues that are required to serve a novel function. 

The *APOLLO* gene was initially described in *A. thaliana* as an exonuclease, protein NEN3, Q9CA74 in Uniprot database [42], probably involved in enucleation of sieve elements, whereas two other copies were described as NEN1 (Q9FLR0) and NEN2 (Q0V842). Given that, we may suggest an evolutionary scenario where, after the series of duplications, one of the NEN protein copies in the common ancestor of *Boechera* spp. might have acquired alter regulation, and might induce development of the apomictic reproduction from the ancestral “sexual” state, following by separation of the apomictic lineages.

That could explain the phenomena of the diploid apomictic *Boechera*, emerged as a result of duplication events rather than polyploidy.

### 3.8. Whole-Genome Comparison

As an example of whole-genome comparison a Circos plot was built for *B. retrofracta* and *B. stricta* (Appendix C). Since both assemblies are performed on a scaffold level, it is difficult to highlight any large genome rearrangements. However, this plot is a visual way to represent the scatteredness of both assemblies.

## 4. Discussion

In this study we present a de novo assembly and annotation of the genome of *Boechera retrofracta*, a perennial flowering plant belonging to Brassicaceae family that is native to North America. The genome of *B. retrofracta* demonstrated a very low level of heterozygosity compare to the genomes of apomictic accessions [2,8,9,10,11,12,13,14,15,16]. Notably, repeats in the genome of *B. retrofracta* occupied almost 40% of the genome space. Nearly half of them were long terminal repeats (LTRs) (18.27%). The genome size was found to be 227 Mb, nearly two-fold larger than the *Arabidopsis thaliana* genome (Table 7). At the same time the amount of protein-coding genes in the genome of *B. retrofracta* is slightly less then in the *B. stricta* and *A. thaliana* genomes and much less than that in the *A. lyrata* genome (Table 1). Despite the largest genome size, the number of predicted transcripts in *B. retrofracta* is the smallest among the four Brassicaceae species compared (Table 1). The presence of a slightly greater number of genes in *B. stricta* compared with *B. retrofracta*, despite a smaller genome size, may be associated with aneuploidy of the chromosomal fragments, or genome rearrangements occurred as a result of interhybridization, which is characteristic of many *Boechera* species and accessions.

As an example of how the genome of the sexual species *B. retrofracta* could be used to study evolution and origin of apomixis, we performed an evolutionary analysis of the three alleles of the *APOLLO* (APOmixis-Linked LOcus) gene (apo- and sex-alleles) described by Corral et al [56]. We examined this gene in more detail in our assembly and in other Brassicaseae species. Along with the described copy of *APOLLO*, we also found two other, more distant copies, which evidently arose by two sequential gene duplications (triplication). The *APOLLO* phylogenetic tree may indicate that triplication event occurred before the separation of Brassicaceae species under study (Figure 4). We also analyzed the *APOLLO* alleles described in *Boechera ssp*. It was clear that these alleles arose after separation of the *Boechera* genus. In sexual *B. retrofracta* and *B. stricta* polymorphic sites corresponded to the “sexallele”-state and clustered with sex-alleles of the other species.

These results are compatible with an evolutionary scenario where, after the series of duplications, one of the NEN exonuclease protein (ancestor of *APOLLO*) copies in the common ancestor of *Boechera* spp. experiencing relaxed selection might be deregulated, promoting development of the apomictic reproduction from the ancestral “sexual” state, following by separation of the apomictic lineages. This model of evolution of *APOLLO* alleles might explain the phenomenon of apomictic development in *Boechera* in the diploid condition, emerged as a result of duplication events rather than polyploidy.

In conclusion, increasing number of sequenced genomes from the economically important Brassicaceae family will facilitate future genetic, genomic, evolutionary, and domestication studies in this family. *B. retrofracta* is thought to be an ancestor of certain hybrids including apomictic species, for example *Boechera divaricarpa*. Consequently, the genome assembly presented in this report may help with the challenging genome assembly of highly heterozygous hybrid *Boechera* species that are apomictic. Thus, the *B. retrofracta* genome reported here will provide a basis to decipher the hybridogenesis events that led to the formation of apomictic *Boechera* accessions.

## Figures and Tables

**Table 1:** General statistics for all stages of the assembly pipeline.

Parameter	Contigs	Extended Contigs	Raw Scaffolds	Intermediate Scaffolfs	Gap Closed Scaffolds	Final Scaffolds
Longest contig	791,985	792,340	8,101,256	9,045,706	9,049,080	9,049,080
Ns	28,100	28,100	11,890,519	16,366,994	12,409,189	12,409,189
Total length	225,649 216	226,402,628	236,469,041	240,945,496	241,014,839	222,253,471

**Table 2:** N50 values for all stages of the assembly pipeline and several different cutoffs for minimal scaffold length.

Scaffold Length Cutoff	Contigs	Extended Contigs	Raw Scaffolds	Intermediate Scaffolfs	Gap Closed Scaffolds	Final Scaffolds
all	85,286	84,648	1,256,534	1,898,006	1,898,985	2,297,899
≥100	85,286	84,648	1,256,534	1,898,006	1,898,985	2,297,899
≥250	101,388	100,393	1,442,421	2,296,484	2,297,899	2,297,899
≥500	115,732	115,486	1,538,795	2,678,857	2,680,941	2,680,941
≥1000	122,300	121,678	1,704,064	2,678,857	2,680,941	2,680,941

**Table 3:** Repeats found by RepeatMasker.

Class	Number of Elements	Total Length (bp)	Fraction of Assembly (%)
SINEs	577	125,298	0.06
LINEs	7075	4,351,241	1.96
LTR elements	51,040	40,608,195	18.27
DNA elements	31,638	12,868,684	5.79
Unclassified	82,693	24,363,135	10.96
Total interspersed repeats	-	82,316,553	37.04
Small RNA	5461	1,599,354	0.72
Satellites	1541	573,026	0.26
Simple repeats	2044	363,642	0.16
Low complexity	56	7456	0

**Table 4:** Results of repeat masking performed by three different tools: RepeatMasker [32], TRF [34], WindowMasker [35].

Tool	Number of Repeats	Total Length (Mbp)
RepeatMasker	173,023	82.31
TRF	100,593	17.41
Windowmasker	1,104,650	64.20

**Table 5:** Annotated transfer RNAs (tRNAs).

tRNA Type	Number
tRNAs decoding standard 20 AA	1126
Selenocysteine tRNAs (TCA)	0
Possible suppressor tRNAs (CTA,TTA)	3
tRNAs with undetermined isotypes	5
Resolution of Brassicaceae Phylogeny Using Nuclear Genes Uncovers Nested Radiations and Supports Convergent Morphological Evolution Predicted pseudogenes	32
Total tRNAs	1166

**Table 6:** Annotated ribosomal RNAs (rRNAs).

rRNA	Complete (≥80% of Expected Length)	Partial (<80% of Expected Length)
5.8S	178	53
5S	601	104
28S	0	1782
18S	1	1458
12S	0	173
16S	0	607

**Table 7:** Comparison of genome characteristics of *Boechera retrofracta* with previously sequenced *Boechera stricta* and *Arabidopsis thaliana* genomes. Source for *B.retrofracta*—this paper, *B.stricta*, *Arabidopsis lyrata* and *A.thaliana*—Phytozome v12.1 database [57].

	*Boechera retrofracta*	*Boechera stricta* v.1.2	*Arabidopsis lyrata* v2.1	*Arabidopsis thaliana* TAIR10
Total length	227 M	184 M	207 Mb	135 Mb
Chromosomes	*n* = 7	*n* = 7	*n* = 8	*n* = 5
Protein-coding loci	27,048	27,416	31,073	27,416
Transcripts	28,269	29,812	33,132	35,386

**Figure C1:**
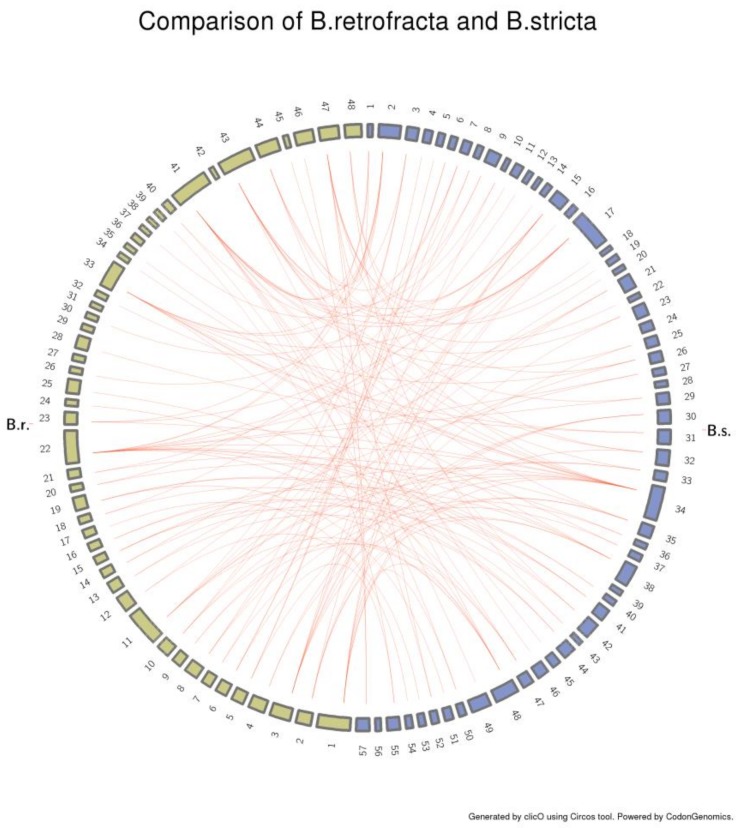
Comparison of *Boechera stricta* and *Boechera retrofracta* genomes on a scaffold level.

## Appendix A

**Table A1:** Sequencing scheme of *Boechera retrofracta* genome.

ID	Library Type	Platform	Read Length	Mean Insert Size (bp)	Number of Reads Pairs
LIB400	paired ends	Illumina	250	402	189788627
LIB4000R	mate pairs	Roche	-	4014	3259085
LIB5000	mate pairs	Illumina	150	4877	19083787
LIB7000	mate pairs	Illumina	150	6882	34066282
LIB24000R	mate pairs	Roche	-	24,332	672098
BES	BAC end sequencing	Sanger	-	147,708	17775

Abbreviations: BAC, bacterial artificial chromosome; BES, BAC end sequencing.

## Appendix B

The original data could be found at: http://public.dobzhanskycenter.ru/ad89dedc8b4674276c9b0760f29b07af/ or at NCBI, BioProject ID: PRJNA418376.
